# Transcriptome Analysis on Monocytes from Patients with Neovascular Age-Related Macular Degeneration

**DOI:** 10.1038/srep29046

**Published:** 2016-07-04

**Authors:** Michelle Grunin, Shira- Hagbi-Levi, Batya Rinsky, Yoav Smith, Itay Chowers

**Affiliations:** 1 Department of Ophthalmology, Hadassah-Hebrew University Medical Center, Jerusalem, Israel; 2 Genomic Data Analysis Unit, Hebrew University, Jerusalem, Israel

## Abstract

Mononuclear phagocytes (MPs), including monocytes/macrophages, play complex roles in age-related macular degeneration (AMD) pathogenesis. We reported altered gene-expression signature in peripheral blood mononuclear cells from AMD patients, and a chemokine receptor signature on AMD monocytes. To obtain comprehensive understanding of MP involvement, particularly in peripheral circulation in AMD, we performed global gene expression analysis in monocytes. We separated monocytes from treatment-naïve neovascular AMD (nvAMD) patients (n = 14) and age-matched controls (n = 15), and performed microarray and bioinformatics analysis. Quantitative real-time PCR was performed on other sets of nvAMD (n = 25), atrophic AMD (n = 21), and controls (n = 28) for validation. This validated microarray genes (like TMEM176A/B and FOSB) tested, including differences between nvAMD and atrophic AMD. We identified 2,165 differentially-expressed genes (P < 0.05), including 79 genes with log2 fold change ≥1.5 between nvAMD and controls. Functional annotation using DAVID and TANGO demonstrated immune response alterations in AMD monocytes (FDR-P <0.05), validated by randomized data comparison (P < 0.0001). GSEA, ISMARA, and MEME analysis found immune enrichment and specific involved microRNAs. Enrichment of differentially-expressed genes in monocytes was found in retina via SAGE data-mining. These genes were enriched in non-classical vs. classical monocyte subsets (P < 0.05). Therefore, global gene expression analysis in AMD monocytes reveals an altered immune-related signature, further implicating systemic MP activation in AMD.

Age-related Macular Degeneration (AMD) is a common degenerative process of the elderly which affects the macula area of the retina. The disease is characterized by degeneration of the retina and retinal pigment epithelium (RPE), and choroid in the atrophic stage of the disease (aAMD). In the neovascular stage of the disease (nvAMD), aAMD is complicated by the growth of choroidal neovascularization (CNV), a condition which often leads to substantial visual loss. Mononuclear phagocytes (MPs), including monocytes and their tissue descendants macrophages, have been implicated in the pathogenesis of both stages of the diseases AMD^1^,^2^,^3^,^4^. MPs are found nearby drusen in aAMD and in the proximity of CNV in nvAMD, and are thought to be recruited from the periphery to the retina, where they may modulate the disease course^3^,^5^,^6^. Macrophages may polarize into variable phenotypes having multiple potential functions, including pro-inflammatory and pro-angiogenic effects^7^. Such effects may be important in the context of aAMD via promotion of RPE and photoreceptor cell death^8^, as well as in the context of nvAMD via enhancement of CNV growth^9^. However, in addition to what is present locally in the retina, evidence shows that systemic inflammation or para-inflammation is present in AMD^2^,^10^,^11^ and might lead to delayed or impaired functions of monocytes and macrophages during aging^4^,^12^,^13^. It is not well established if monocytes present in systemic circulation reflect, modulate, or partially underlie the disease process. Systemic inflammation in AMD is not only supported by tangential biomarkers, but also genetic data^14^, as mutations or variants in genes representing the complement immune system are a risk factors for AMD^15^,^16^. As monocytes interact with chemokine factors released by cells present at inflammatory sites, and as the microenvironment of the retina changes due to inflammation, immune cells, and the possible disease states, the subsequent response may be an important factor in AMD^17^,^18^.

Monocytes can be subdivided into two major subgroups: the CD14++CD16−, or classical monocyte, and the CD14+CD16+, or non-classical subgroup (which can be further subdivided into two groups depending on CD16+ expression). These two subgroups have been found to be similar although not identical in both mouse and in human, and are identified using different markers- F4/80, Ly6C/G, CD11b, and MHCII in mouse, while CD14 and CD16 along with HLA-DR and CX3CR1 in human^19^,^20^. The CD14+CD16+ subgroup has been known as the monocytes that migrate to sites of injury and inflammation or that are active during a disease state^19^,^21^,^22^,^23^. We previously identified altered gene expression in peripheral blood mononuclear cells (PBMCs), a cell population which includes monocytes^24^, and increased expression of major chemokine receptors CCR1 and CCR2 in the CD14+CD16+ subset of monocytes in nvAMD patients^2^. Differential expression of additional proteins related to immune responses such as CD46, CD59 and CD200 was reported in white blood cells from AMD patients^17^,^25^.

While these data suggest monocyte involvement and not only macrophages in AMD, a comprehensive view on monocyte involvement in AMD is still lacking. Analysis may provide important insights to AMD pathogenesis, as well as clarifying if monocytes can be regarded as a target/biomarker for the disease or progression. Therefore, we wished to investigate for the first time the global gene expression of blood monocytes from patients with nvAMD and age-matched controls via microarray analysis.

## Results

### Microarray Bioinformatics

**Figure 1:**
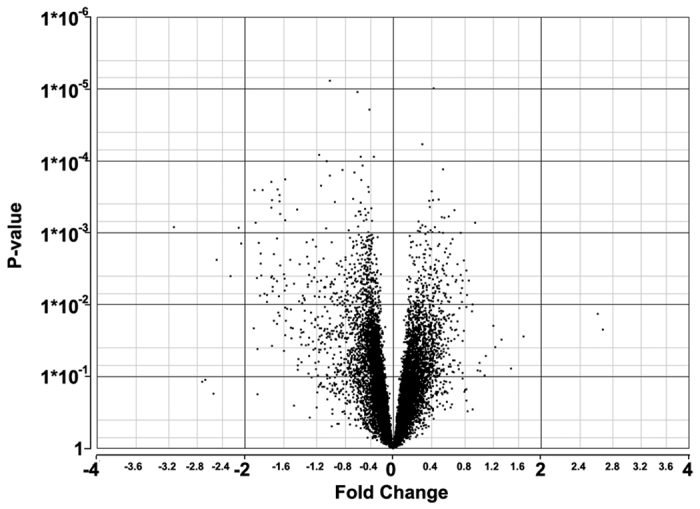
Volcano plot (P-value vs. Log2 Fold Change) of all genes present in microarray comparison between nvAMD patients and controls. Positive Fold Change (log2 FC > 0) indicates upregulated in nvAMD monocytes, negative fold change (log2 FC < 0) indicates downregulated in nvAMD. X axis shows log2 FC level, Y axis shows significance level.

To investigate monocytes’ role in AMD, we performed whole genome microarray analysis on patients with nvAMD. We chose nvAMD patients as they represent the most aggressive form of the disease. All patients included in the study also had manifestations of aAMD. Our results via RMA (Robust Multi-array Average) analysis and ANOVA (analysis of variance) testing^26^ via R (https://cran.r-project.org/ and http://www.rstudio.com), and Partek Genomics Suite (Partek, St Louis, MI, USA) indicated that 2,165 genes were differentially-expressed (both up and downregulated genes) on mRNA from blood monocytes between treatment-naïve nvAMD patients and age-matched controls (P < 0.05, ANOVA; found in Supplementary Material, Table S1). Of those genes, 79 genes had an absolute fold change (log2 FC) >1.5 difference between AMD and control monocytes (indicating both upregulated and downregulated genes), and 506 genes had a absolute log2 FC >1.2 (Table S1, Table 1 and Fig. 1). We followed up with RMA analysis via EXPANDER (EXPression Analyzer and DisplayER, http://acgt.cs.tau.ac.il/expander/overview.html) program^27^, which validated the results previously found via Partek’s algorithm (431 differentially-expressed genes were identical with P < 0.05 in both datasets, found in Supplementary Material, Table S2). Differentially-expressed genes were identified by EXPANDER as upregulated in nvAMD (n = 229) or downregulated (n = 370). Further analysis with GSEA’s (Gene Set Enrichment Analysis, http://www.broadinstitute.org/gsea/index.jsp)^28^ algorithm to identify differentially-expressed genes and enrichment of genes or curated gene lists between two phenotypes confirmed the top expressed genes (which included 70/506 genes with log2 FC > 1.2 in both datasets) both up and downregulated in the microarray between nvAMD and controls (found in Supplementary Material, Fig. S1 and Table S3).

### QPCR

We used monocyte mRNA from treatment-naïve nvAMD patients (n = 25), aAMD patients (n = 21), and age-matched examined controls (n = 28) that were not used for microarray for validation via QPCR experiments. Including both stages of the disease (aAMD and nvAMD) allowed us to investigate differences between them. Six genes of interest: TMEM176A, TMEM176B, OLR1, FOSB, FAIM3, and MS4A1, chosen from the top genes based on their magnitude and significance of expression in nvAMD were tested (P < 0.05, log2 FC > 1.5. TMEM176A/B, OLR1, and FOSB were upregulated in nvAMD, while FAIM3 and MS4A1 were downregulated).

**Figure 2:**
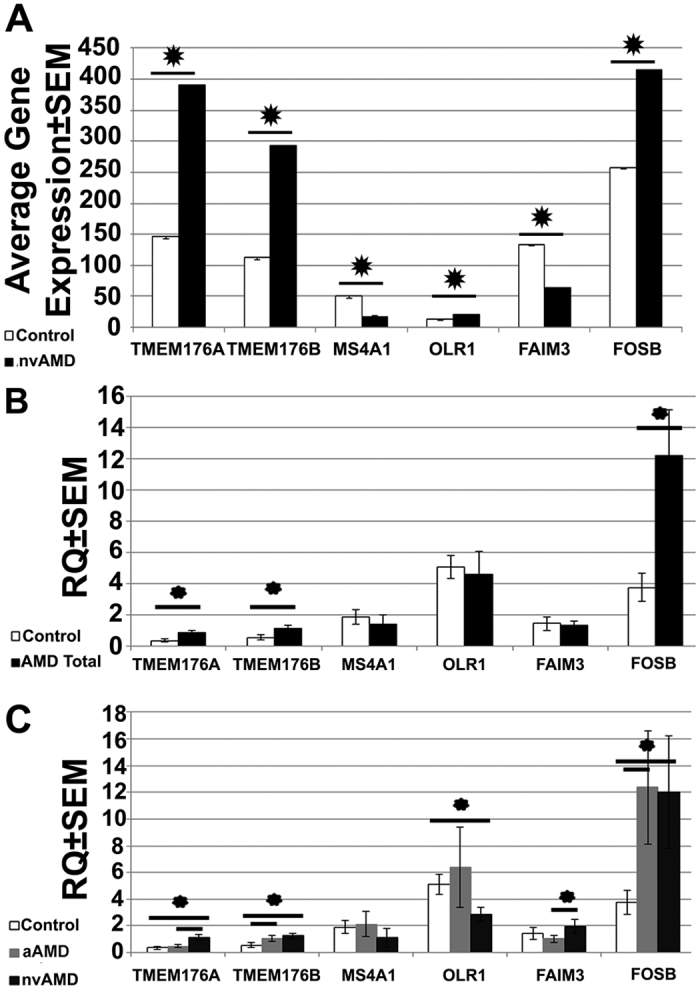
Differential expression of genes in nvAMD monocytes according to microarray analysis and QPCR. Panel A indicates average log2 gene expression ± SEM from RMA-normalized microarray results for comparison with QPCR. QPCR results for six differentially expressed genes in nvAMD monocytes (P < 0.05, log2 FC > 1.5) tested on nvAMD and aAMD patients and controls not previously used for microarray data. Panel B indicates general comparison between total AMD patients and controls; panel C indicates separation between nvAMD, aAMD, and controls. * = P < 0.05.

Four comparisons were evaluated: nvAMD vs controls (1), aAMD vs controls (2), total AMD vs. controls (3), and nvAMD vs aAMD (4). QPCR results validated the microarray analysis (Fig. 2A) for five of the six genes tested (Fig. 2B). TMEM176A, TMEM176B, FOSB, and OLR1 were upregulated in nvAMD vs controls (P < 0.05), while TMEM176B and FOSB was upregulated in aAMD vs controls (P < 0.05). TMEM176A, TMEM176B and FOSB were upregulated also in total AMD patients vs controls (P < 0.05). TMEM176A and FAIM3 showed upregulation in nvAMD vs aAMD (P < 0.05) (Fig. 2C). OLR1 was downregulated in nvAMD instead of controls different to microarray.

### Pathway and Functional Analysis

**Figure 3:**
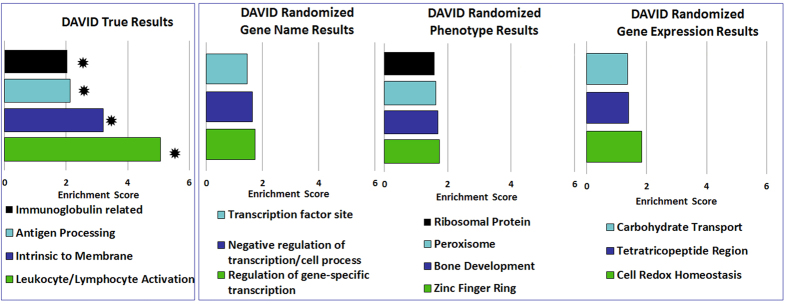
DAVID results from RMA-normalized data’s gene list (N = 2,165) indicating enriched clusters (EC): (FDR-P < 0.05, EC > 2) as compared to randomized data results performed in three randomization techniques: randomized gene names, randomized phenotype, and randomized gene expression results. Randomized results showed no significance (P > 0.05, EC < 2). * indicates P < 0.05.

We used DAVID’s algorithm (Database for Annotation, Visualization, and Integrated Discovery, http://david.abcc.ncifcrf.gov)^29^ for pathway/functional analysis to evaluate all 2,165 differentially-expressed genes. Fourteen main annotation clusters were associated with the differentially-expressed genes (P < 0.05, 4/14 clusters had an FDR-P (false discovery rate-P) < 0.05, Enrichment Score (ES) >1.5). Among these clusters, seven were related with immune system or inflammation (found in Supplementary Material, Table S4). In DAVID, all randomized datasets (name, phenotype and gene expression) did not show significant clusters (FDR-P > 0.05, ES < 1, Fig. 3).

To validate DAVID’s findings, we performed additional functional annotation analysis via the Enrichr program (http://amp.pharm.mssm.edu/Enrichr/)^30^ exactly similar to the DAVID methodology. Results were highly similar, with the top ranked GO terms being leukocyte activation, lymphocyte activation and immune response for all sets of data tested via the various fold change sets (adjusted-P < 0.05, Enrichr algorithm, Table S5). Randomized data analyzed in the same way as performed with the DAVID algorithm did not show the same clusters or significance levels as actual data analysis.

**Figure 4:**
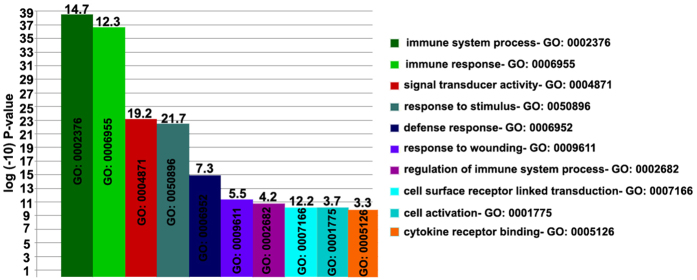
Functional analysis of differentially expressed genes in nvAMD monocytes. TANGO results for GO clusters from differentially expressed genes (N = 2,165, P < 0.05), indicating the top 10 clusters found. -log10 P-value is present as the Y axis, developed according to TANGO’s bootstrap algorithm. The number on top of each cluster’s column indicates the percentage of differentially expressed genes (2,165 with P < 0.05 via RMA analysis) present in the functional class cluster (see results sections for more details). Complete results of 29 functional clusters enriched in nvAMD monocytes are presented in Fig. S2.

Similarly, another GO (Gene Ontology, http://geneontology.org) analysis program, TANGO from the EXPANDER program, was used on RMA-normalized data, and found 29 differentially-expressed clusters (FDR-P < 0.05) via the 2,165 differentially-expressed genes. Both algorithms identified variations on the ‘immune system process’ term as highest-ranked cluster. TANGO identified “defense response” as another highest cluster (P < 0.05, Fig. 4 and in Supplementary Material, Fig. S2).

DAVID was used to evaluate 506 genes that had a log2 FC of 1.2, and the main annotation clusters can be found in Supplementary Material, Table S4 available online (25 clusters FDR-P < 0.05, ES > 2.5). In accordance with the results of the analysis of the 2,165 genes, 16/25 clusters were related with immunity or inflammation.

When we applied DAVID on RMA-normalized data from 79 genes with log2 FC > 1.5 between AMD and controls, we identified 10 annotation clusters that were upregulated (FDR-P < 0.05, EC > 2). These included multiple clusters associated with immune response such as: membrane-bound, immunoglobulin like, antigen processing, lymphocyte/leukocyte activation, immune response activation, and T-cell receptor pathways (Supplementary Material, Table S4). These results indicated the presence of an inflammatory/immune signature relating to the disease.

To exclude that the association of immune genes with AMD was by chance, all 20,364 genes present on the microarray chip were visualized via DAVID, and 254 genes were identified as relating to the GO term immune response (GO: 0006955). DAVID identified 24/79 differentially-expressed genes as linked to that GO term (χ2 test P < 0.0001). This suggests that the 79 genes associated with AMD are enriched for immune response genes compared with the representation of such genes on the microarray platform.

### Comparing AMD Monocyte Gene Expression Signature across Datasets

Our GSEA analysis identified nine enriched (P < 0.05) lists upregulated in AMD patients (Normalized Enrichment Score (NES) >1.5, 6/9 of which FDR-P < 0.05) and six lists enriched in genes downregulated in nvAMD (NES >1.5, 5/6 of which FDR-P < 0.05) (Supplementary Material, Table S6). Enriched lists included genes involved in dendritic cell maturation (2.4 ES, P = 0.002), or inflammatory response to LPS (lipopolysaccharide) (2.6 ES, P = 0.002), which were upregulated in genes that were also upregulated in nvAMD compared to controls.

We used all genes at different significance levels (2,165, 506, and 79) in an unbiased search with GSEA/MSigDB for Categories 1:7 (C 1:7). This showed other enriched gene lists (P < 0.05), including genes upregulated in lupus, genes involved in monocytes connected with HIV or influenza, or other immune responses (Supplementary Material, Table S7).

### Analysis of Immune Gene Expression Datasets

We compared our microarray data with publicly available GEO microarray data on human controls from Zeigler-Heitbrock *et al*.^23^, (ZH) which showed differentially-expressed genes between two main human monocyte subsets (CD14++CD16- and CD14 +CD16+). There were 981 differentially-expressed genes between the two subsets (P < 0.05). Of the differentially-expressed genes in ZH’s dataset, 139 were also differentially-expressed in nvAMD (χ^2^ < 0.0001). With our data, 20 of the 79 log2 FC > 1.5 genes were found differentially-expressed amongst monocyte subsets (χ^2^ = 0.04), indicating certain genes may be active in monocyte subsets, and between nvAMD and controls.

We subdivided the genes from the ZH dataset into upregulated in non-classical monocytes versus upregulated in classical monocytes. Of 981 genes, 351 genes were upregulated in non-classical monocytes, and 384 were upregulated in classical monocytes (P < 0.05, log2 FC > 1.2). We then compared the 506 genes from our dataset that had a P < 0.05, log2 FC > 1.2 to the ZH dataset of differentially-expressed genes. Of the genes differentially-expressed in nvAMD, 13.6% were upregulated in non-classical monocytes, while only 6% were upregulated in classical monocytes (P = 0.001; χ^2^).

We utilized the Immunological Genome Project (ImmunGen, https://www.immgen.org)^31^ to compare genes expressed in several monocyte subsets, Four comparisons were made: between MHC+ subsets and the microarray data, and the MHC- subsets and the microarray data. Between the MHC- mouse datasets, 282 genes were higher in non-classical monocytes, while 659 were higher in classical (P < 0.05, log2 FC > 1.2). Compared to the 506 microarray genes (P < 0.05, log2 FC > 1.2), 26 genes (5%) overlapped with the classical subset while 20 genes (4%) overlapped with the non-classical (P = 0.003; χ^2^). Of the 79 genes with log2 FC >1.5, 7 genes (8%) overlapped with the non-classical subset while 4 (5%) overlapped with the classical (P = 0.02; χ^2^). Between the MHC+ mouse datasets, 187 genes were higher in non-classical monocytes, while 783 were higher in classical (P < 0.05, log2 FC > 1.2). Compared to the 506 genes, 5 (0.08%) overlapped with the non-classical subset, while 43 (8%) overlapped with the classical (P = 0.18; χ^2^). Of the 79 genes with log2 FC >1.5, 3 genes (3%) overlapped with the non-classical subset, and 0 with the classical (Supplementary Material, Table S8-10).

We evaluated SAGE (Serial Analysis of Gene Expression) data from the National Eye Institute (NEI, http://neibank.nei.nih.gov/EyeSAGE/index.shtml)^32^,^33^ for expression in control retina, macula, and body tissue for the top ranked genes of the 79 enriched genes (P < 0.05, log2 FC > 1.5) (Table 1). All genes tested had at least 3 tags with >95% accuracy. The data showed that all genes investigated had a significant presence (>10 tags) in control human retina, and in macula (>5 tags). TMEM176A/B and FOSB had >10 tags in macula. Tags present in retina, RPE and other ocular data from the UCSC Browser (https://genome.ucsc.edu)^34^ and the Ocular Genomics Institute data indicated transcripts present in control retina (Supplementary Material, Table S9).

### Regulatory pathways analysis

We wished to evaluate transcription factors (TFs) and TF families, along with miRNAs (microRNA) that may regulate the genes in AMD monocytes. We used binding motif and seed analysis known for TFs/miRNAs, using ISMARA (Integrated Motif Activity Response Analysis, http://ismara.unibas.ch/fcgi/mara)^35^ to evaluate for TFs that explain the changes in transcription levels between AMD and controls. Several TFs motifs were enriched in differentially-expressed genes. The enriched TFs in genes most upregulated in nvAMD patients was the AHR family of TFs (ES = 0.66), involved in regulation of angiogenic factors, including vascular endothelial growth factor-alpha (VEGF-α). The most enriched TF in genes downregulated in nvAMD patients was AIRE (ES = 0.58), an autoimmune regulator involved in avoidance of immune reactions, and mutations in which cause immune diseases (P < 0.05, ES > 0.2). Another enriched TF in genes differentially-expressed in AMD, HLF (ES = 0.384), has a main target in interleukin-1-beta, which is a pro-inflammatory factor.

Multiple miRNA motifs were also enriched (ES > 0.2). Downregulation of the hsa-miR-383 (miRNA 383, ES = 0.43, ISMARA) was detected in nvAMD patients. One of miRNA 383’s targets, according to Tarbase, is VEGF- α. Potentially, repression of VEGF- α via hsa-miR-383 in controls exerts a protective effect (Supplementary Material, Fig. S3).

GSEAs algorithm was applied in an unbiased search of MSigDB for the three levels of significance of differentially-expressed microarray genes in the TF/miRNA collection list (C3, Supplementary Material, Table S10). This analysis identified the highest gene enrichment (P < 0.05) of differentially-expressed genes (268/2065) that had the binding motif CAGGTG for the TF TCF3, an autoimmune regulator (FDR-Q value = 3.33 ^e−^38), indicating that this TF may be involved in regulating gene expression, involving a dysregulation of the immune system.

We used UCSC Genome Browser^36^ in combination with the MEME suite (http://meme-suite.org)^37^ to identify enriched motifs in transcripts from genes upregulated or downregulated in AMD compared to shuffled data. Highly-enriched TFs in AMD patients were the IRF family and the MEF family, while highly-downregulated TFs in nvAMD included HSF1 and ZIC2. This analysis also validated all TFs found in the earlier ISMARA analysis, which was based on the JASPAR and Transfac database, with the MARA algorithm for TFs enriched between AMD and controls (Supplementary Material, Table S11).

### Comparison of Expression Levels to Clinical Parameters and Genetics

We investigated clinical, demographic and genetic data to see if any parameter affected gene expression. In samples that underwent QPCR, we found an association between upregulation of TMEM176B and the presence of a risk allele of C3 in controls (P = 0.03), as well as upregulation of FAIM3 and the risk allele of C3 in all AMD patients, both atrophic and neovascular (P = 0.02). No other associations were found between mRNA levels in patients or controls. Yet, the power of these comparisons was smaller than 80%.

## Discussion

AMD, despite being an ocular disorder, may contain, as shown by our results, a systemic inflammatory signature that is found in monocytes from patients with both the atrophic and the neovascular stages. This was shown using microarray analysis and bioinformatics data on patients with a combination of aAMD and nvAMD, and validation via QPCR on additional groups of patients with either aAMD or a combination of aAMD and nvAMD. Genes like TMEM176A/B and FOSB showed upregulation in both stages of AMD, while genes like FAIM3 were upregulated in nvAMD as compared to aAMD. Several pathways, among them defense mechanisms, receptors and responses to stimulus, and inflammation or immune responses, which are upregulated in other systemic inflammatory disorders, are altered in AMD monocytes^38^.

Of particular interest among the differentially-expressed genes are the TMEM176A/B genes. These genes are 97% identical, (ClustalW, http://www.ebi.ac.uk/Tools/msa/clustalw2; BlastP,  http://blast.ncbi.nlm.nih.gov/blast/Blast.cgi?CMD=Web&PAGE_TYPE=BlastHome; and Uniprot, http://www.uniprot.org) and evolutionarily compromise a family, found in mammals and boney fish^39^. Little is known about these genes, who play possible roles in the maturation of dendritic cells and MPs^40^, but recent research has shown involvement in cancer angiogenesis^41^, and that they are tolerance proteins whose upregulation and function in controlling ionic homostasis predicts better response to allographic transplantation^42^. In fact, TMEM176 genes may be involved in macrophages and Alzheimer’s disease, in processing of lipid-plaques, where TMEM176+ cells were least able to clear plaques, causing increased apoptosis^43^.

Mice with a targeted knockout (KO) of TMEM176B seem to have a smaller cerebellum indicating involvement in brain development. Cerebellar granule cells, most similar to retinal neurons, were found to be developmentally dependent on TMEM176B^44^,^45^. The BrainStars database (http://brainstars.org)^46^, along with the Allen Brain Atlas (http://www.brain-map.org)^47^ indicate that TMEM176A/B are highly expressed in mouse retina. The upregulation of these genes may indicate a tolerance-mediated response to the inflammation of AMD. According to this hypothesis, systemic para-inflammation of AMD may cause tolerance-related genes in cells involved in the disease, namely, monocytes, to be upregulated to cope with the disease burden. In accordance with that, upregulation of TMEM176A/B was reported in immature dendritic cells^40^. Furthermore, we compared gene expression in monocytes stimulated with GMCSF and IL4^48^ (that became dendritic cells) to gene expression in nvAMD monocytes and found similarities in the gene expression patterns. Taken together, these data suggest that while activation of monocytes may occur in AMD patients, multiple feedback mechanisms may be in place to regulate or suppress their activation and subsequent differentiation to macrophages in retinas affected by aAMD and nvAMD.

Monocytes may also be associated with angiogenesis caused by their descendants, macrophages, recruited from the periphery to the eye. However, they may have the third function, regarding macrophages’ response to AMD. Upregulation of TMEM176A/B in Alzheimer’s disease was attributed to lower/less effective functioning of macrophages in lipid clearance. Similar process, occurring in aging, with some genetic predisposition, and alteration of macrophages’ key actions possibly in association with TMEM176A/B dysregulation, may be part of AMD pathogenesis.

Another gene of interest upregulated in AMD monocytes according to our research is FOSB. The product of this gene is part of the TF AP-1, which is involved in inflammatory pathways, including the NFK-B pathway that is involved in apoptosis, angiogenesis, and inflammatory response^49^. AP-1 is a TF that is comprised of heterodimers of c-Jun, JunB, or JunD, with c-Fos, FosB, Fra1, and Fra2^50^. c-fos, which is a component of AP-1, is necessary for light-induced damage and photoreceptor apoptosis, and KO c-Fos mice are resistant to light damage. Therefore, upregulation of another component of AP-1, which is part of the dimeric complex, may indicate the initiation of damage processes/apoptosis^51^. AP-1 is a regulator of gene expression in situations of oxidative stress^52^. In monocytes specifically, upregulation of cytokines and tissue factors of inflammation has caused upregulation of AP-1, and NFK-B activation, indicating a possible mechanism for upregulation of FOSB in AMD monocytes^53^. Upregulation of FOSB was also found in aged macrophages from mice, along with the anti-inflammatory phenotype of those macrophages, which in the same study had increased IL10 and increased CNV^54^.

A small difference in the ages exists between the AMD patients examined here and the controls (AMD average age: 76.51 ± 1.35, control average age: 71.86 ± 1.07). Such a small absolute numeric difference compared to the value for age itself is unlikely to bias the findings of this research. It is interesting also to note that other studies evaluating the transcriptome of human retinal cells, such as RPE, or animal models resembling some features of AMD, have been reported, but show no overlap between their top differentially expressed genes and our study’s top genes^55^,^56^. Yet, these previous studies did not focus on immune cells.

Monocytes, as well as being ambassadors of immune responses and defense mechanisms, were implicated in AMD pathogenesis^2^,^8^,^18^, and the results here indicate that this occurs on a whole genome level. It is unclear if altered monocyte gene expression in AMD is primary or secondary to the disease process as the disease state does change the microenvironment, and in turn, potentially, the expression profile^57^. Other inflammatory disorders such as: heart failure^38^, kidney disease and rheumatoid arthritis^58^ all demonstrate transcriptome changes in monocytes. In a state of inflammation, monocytes respond and are recruited to inflamed areas, but also adopt specific microenvironment signals to transform into polarized macrophages once inside tissues. These polarized macrophages were implicated in the pathogenesis of both aAMD and nvAMD^4^,^59^. Therefore, the signals here present in the transcriptome may be part of a general mechanism of reaction towards either chronic inflammation, or a general recruitment to the source of inflammation itself^3^,^60^.

We have examined the total blood monocyte population from patients with nvAMD, and have shown a differential inflammatory response present in their transcriptome when compared with control blood monocytes. This study further indicates that monocytes play a role in AMD. It is still unclear whether monocytes participate in modulating or antagonizing the disease state. These findings strengthen the notion of para-inflammation or a systemic inflammatory state in AMD. Further research targeting monocyte recruitment, differentiation to macrophages while in ocular environment, and possibly eventual cell therapy or reprogramming, may be worthwhile investigations to pursue in the future.

## Methods

### Patients and Controls

Treatment-naïve nvAMD patients (n = 14, female/male: 6/8, average age: 79 ± 2.05 years, range: 63–89) were recruited from the retina clinic of the Department of Ophthalmology at the Hadassah-Hebrew University Medical Center. Criteria for inclusion of nvAMD patients included: age >60, AMD diagnosis according to AREDS (Age-Related Eye Disease Study)^61^, and CNV diagnosis according to fluorescein angiogram (FA). Eyes with neovascular lesions comprised of <50% active CNV, subretinal hemorrhage >25% of lesion size, or presence of other retinal diseases were excluded. Specifically, eyes with other potential CNV causes such as myopia, trauma, or uveitis were excluded. Also excluded were patients with major systemic illness, such as cancer, autoimmune disease, congestive heart failure or uncontrolled diabetes.

Age-matched controls (n = 15, female/male: 6/9, average age: 72 ± 1.79 years, range: 60–84) were recruited from the same clinic. Inclusion criteria were: age >60, no known retinal diseases or glaucoma, no recent eye surgery, with unremarkable ophthalmoscopy, and no systemic illness. Controlled diabetes mellitus (DM) incidence was similar between AMD patients and controls (9/60 AMD patients with DM, 10/42 controls with DM, χ^2^ = 0.86).

Demographics, medical and ophthalmic history, and findings from ophthalmic exams were collected. Optical coherence tomography (OCT) and FA images from the nvAMD patients were reviewed.

An additional set of nvAMD patients (n = 25, female/male = 16/9, aged 75.78 ± 2.35; range 60–96), aAMD (n = 21, female/male = 11/10, aged 74.56 ± 2.56; range 54–84), and age-matched controls (n = 28, female/male = 17/11; aged 71.93 ± 1.33; range 61–89), who were not used for microarray, were recruited for quantitative real-time PCR (QPCR) validation experiments.

Ethical approval for all experimental protocols and study were approved by the local Committee on Research Involving Human Subjects of the Hebrew University-Hadassah Medical School, the Helsinki Committee of Hadassah Medical Organization, and the Israel Ministry of Health’s Helsinki Committee for Genetic Experiments on Human Subjects. All patients and controls signed informed consent forms that adhered to the tenets of the declaration of Helsinki before participating in the study. All methods used in the study were carried out in accordance with the approved guidelines for the study.

### DNA extraction and genotyping

Blood samples (2 ml) from patients and controls were collected into EDTA-containing vaccutainer tubes. DNA extraction was performed using the FlexiGene kit (Quiagen, Hilden, Germany) with 300 microliter (ul) of blood according to manufacturer’s instructions. Concentration and quality were checked via Nanodrop (Thermo Scientific, Waltham, MA, USA). DNA was stored at −20 °C.

Patients and controls were genotyped for three main SNPs associated with AMD in Israeli populations as previously described^2^ (rs11200638 in the intron of HTRA1, rs1061140 in CFH, and rs2230199 in C3). Polymerase chain reaction (PCR) were performed as previously described using PCR Master Mix (Kapa, Wilmington, MA, USA), and primers (Sigma-Aldrich, Munich, Germany) designed via UCSC Genome Browser, NCBI or Primer3 and evaluated on 1.5% agarose gels using nucleic acid staining solution (RedSafe, iNtron Biotechnology, Summit, NJ, USA). PCR reaction products were sent to sequencing (Macrogen, South Korea) and results were interpreted using Chromas (http://technelysium.com.au/?page_id=13, South Brisbane, Australia) or Gap (http://staden.sourceforge.net, Staden Package, Cambridge, England).

### RNA extraction and microarray analysis

RNA extraction was performed as previously described^2^. Briefly, 15–20 ml of fresh blood was collected in EDTA-containing tubes. Samples were processed within 6 hours. Blood was centrifuged on a Ficoll (Sigma) gradient to remove granulocytes, and the PBMC layer was isolated. PBMCs were washed twice with RPMI medium to remove platelets, and cells were counted using a hemocytometer after staining with Trypan blue to exclude dead cells. Over 2.5 × 10^7 cells were used per experiment for greatest yield. Cells were spun down and resuspended in monocyte isolation buffer (2% FCS, 1 mM EDTA in filtered PBS) according to manufacturer’s instructions, and monocytes were isolated with magnetic beads (EasySep Kit #19058, Stemcell Technologies, Vancouver, Canada). This selection kit was used because of negative selection properties, to prevent potential interference with RNA expression in cells positively labeled with antibodies. Successful monocyte isolation was validated using flow cytometry following staining with CD14 and CD16 as described previously^2^. We normally obtained around 5 × 10^5 monocytes per sample. Monocytes were spun down and immediately suspended in 1 ml of Trizol (Sigma). RNA extraction was performed using TriReagent (Sigma) according to manufacturer’s protocol. RNA was incubated at 55 °C for 10 minutes and treated with DNase. RNA underwent further cleaning (Qiagen MiniElute kit) according to manufacturer’s protocol, was suspended in 14 ul of DEPC-treated water, and frozen at −80 °C. RNA quality and quantity was evaluated using NanoDrop spectrophotometer and using Agilent Bioanalyzer. Only monocyte RNA with an RNA Integrity Number (RIN) greater or equal to 8 was used for microarray or QPCR. RNA was converted to cDNA and hybridized to Affymetrix Human Gene 1.0 ST microarrays (GeneChip, Affymetrix, Santa Clara, CA) according to manufacturer’s instructions. The process was as follows: Amplified and biotinylated sense-strand DNA was prepared according to standard Affymetrix protocol from 200 ng total RNA (Expression Analysis Technical Manual, 2005, Affymetrix). Following fragmentation, 5.5 μg of Biotinylated ssDNA were hybridized for 17 hrs at 45 °C on the GeneChip Array. The GeneChips were washed and stained in the Affymetrix Fluidic Station 450. GeneChips were scanned using Affymetrix GeneChip Scanner 3000. Hybridization took place in batches, and batch effect was taken into account for downstream analysis. MIQC/MIAME guidelines were followed^62^.

### QPCR

RNA from AMD patients (aAMD = 21, nvAMD = 25) and controls (n = 28) who did not undergo microarray analysis and fulfilled the same inclusion/exclusion criteria as the samples used for microarray was processed in the identical way as microarray RNA. RNA was reverse-transcribed to cDNA using the High Capacity Reverse Transcription Kit (Applied Biosystems, Carlsbad, CA, USA) according to manufacturer’s protocol, and cDNA was stored at −20 °C. TaqMan gene expression assays (Applied Biosystems) for the genes TMEM176A (Assay ID: Hs00962650_m1), TMEM176B (Hs00218506_m1), FOSB(Hs00171851_m1), OLR1(Hs01552593_m1), FAIM3(Hs00193770_m1), and MS4A1(Hs00544819_m1), and endogenous gene RPLP0 (Hs99999902_m1), chosen because of its strength in analyzing PBMC, were used according to manufacturer’s protocol. Accuracy of QPCR primers was checked via successive dilution of representative cDNA samples. QPCR was performed in triplicates using StepOne Plus (Applied Biosystems) machine, analyzed using StepOne Software vs2.2, DataAssist v2.0 (Applied Biosystems) and spreadsheet software (Excel, Microsoft, Redmond, WA, USA). All gene expression data was analyzed according to the 2-delta-delta CT method.

### Bioinformatics analysis

#### R/Bioconductor and Partek

Raw cel-file (.CEL) data was analyzed using R, R Studio, and Bioconductor (http://www.bioconductor.org), as well as Partek Genomics Suite. We utilized multiple R packages for the purpose of the study including affy^63^, oligo^64^, limma^65^, simpleAffy^66^, and oneChannelGUI^67^ (R codes are available upon request from the authors). RMA was used for the normalization method, and after RMA, all values continued in log2 transformation. Quality control based on array, genes, and sample was performed to evaluate for biased arrays. Batch effect was taken into account for analysis, as was gender and sample type. ANOVA was used for multivariate comparison on all genes and arrays, and correction for multiple comparisons was applied via FDR^26^. Genes were considered significant if P < 0.05 after multiple comparison correction and batch effect was applied, and fold change was applied on log2 values. Therefore, differentially expressed genes had a log2 FC greater or less than 1. RMA-processed data was used for downstream analysis and statistics unless the program used requested a start of CEL data. EXPANDER^27^ was used to process. CEL data as well, using RMA analysis to obtain results for differentially expressed genes^68^,^69^.

#### GO/KEGG analysis

DAVID analysis^29^ was applied to genes that were differentially-expressed between nvAMD and controls. This used all genes for analysis that had p-value < 0.05, as well as separating the significant genes into sets with different FC (log2 FC = 1.5, log2 FC = 1.2). Randomized sets were generated from microarray data and used as controls [randomized name, phenotype (AMD/control), expression levels culled from microarray raw or RMA-adjusted results]. To validate the association of functional classes identified by DAVID, several randomized datasets, each encompassing 10 permutations of randomization algorithms, were used to validate the null hypothesis. This was done in three different fashions using each of the three randomized sets. Microarray genes in total were evaluated based on GO terms^70^. Significant clusters had a FDR-P < 0.05. This approach was validated with three other GO analysis programs: Enrichr (which included GO, Reactome, KEGG, and Proteome Tissue Expression, shown in Supplementary Table S5, TANGO^27^ and Princeton GO analysis program.

#### Gene Set Enrichment Analysis (GSEA)

GSEA^28^ and MSigDB^71^ were utilized for two-step testing. Firstly, RMA-normalized data was used to validate gene expression and differentially-expressed genes. Secondly, to determine genes that were differentially- expressed and enriched compared to curated or non-curated (over all 5 categories which GSEA provides) gene lists via GO terms or via experimental data from MSigDB. Gene lists were pulled from MSigDB to determine enrichment of genes in 49 predetermined lists (Supplementary Material, Table S6 . Molecular Signatures These lists are based on GO, KEGG (Kyoto Encyclopedia of Genes and Genomes, www.genome.jp/kegg) and peer-reviewed data that related to monocytes, inflammation, macrophages, retina, and associated pathways. In addition, enrichment search was performed using GSEAs unbiased search which looks at curated gene-lists, and also at TFs and miRNAs whose binding motifs are enriched in differentially-expressed genes, or whose general signature of downstream genes/pathways are affected.

#### Motif analysis

Identification of AMD-associated motifs was performed using ISMARA^35^. Analysis was performed on raw. CEL data to compare differentially-expressed genes between nvAMD and controls. It looked at genes upregulated and downregulated in nvAMD separately for each analysis. It was then used to determine TF and miRNA motifs that may control/affect expression. It was able to infer activities of TFs/miRNAs across the samples based on raw data, and interactions between regulators. TF analysis was performed in MARA using motifs from JASPAR (http://jaspar.binf.ku.dk)^72^ and Transfac (http://www.gene-regulation.com/pub/databases.html). The miRNA signature found by ISMARA was looked at using a combination of mirBase^73^ and Diana’s Tarbase (http://diana.imis.athena-innovation.gr/DianaTools/index.php?r=tarbase/index)^74^. Results were used as a springboard to infer global interactions different between patients and controls.

Motif analysis was also performed using a combination method. Using UCSC Genome Browser^34^, we pulled 700 base pairs before the coding region of the top 50 genes and the 506 genes with a log2 FC > 1.2 (up and downregulated) to investigate TFs. This was done separately for genes upregulated and those downregulated in AMD, to investigate TFs working individually. Each data set was investigated using the MEME suite (http://meme-suite.org)^37^. The AME tool was used to investigate enriched motifs in transcripts upregulated or downregulated in AMD compared to shuffled data. The HOCOMOCO database was used for identification of TF motifs. TFs that were found in both upregulated and downregulated genes were eliminated from analysis and TFs were considered enriched if found not only significant (P < 0.05) via MEME, but also if found in comparison between upregulated and downregulated genes and between the respective data set and its shuffled data (Supplementary Material, Table S11).

#### Immunological and retinal gene expression evaluation

We used ImmuGen^31^, which allows visualization of populations from human and mouse samples, as well as separate populations of monocytes (CD14+CD16- and CD14+CD16+ subsets), for further comparison to microarray data. These cells are taken from mice from different bodily locations with different immunological profiles. Data from subsets of monocytes (mouse, sorted via flow cytometry into several subsets, including classical and non-classical) was examined. ImmuGen mouse blood monocytes are sorted based on a MHCII+ or–basis.We evaluated for enrichment of the genes identified in microarray analysis in one specific cell population. Data publicly available via GEO from Ziegler-Heitbrock, *et al*.^23^, (GEO: GSE34515, ZH) was normalized and analyzed with R using the same analysis pipeline described above. ZH data was used as a baseline to evaluate differentially-expressed genes from microarray in comparison with the CD14-CD16 subsets in healthy individuals.

The National Eye Institute (NEI) EyeSage database^32^,^33^ was used to compare differentially-expressed genes with gene expression in control human retina and macula. Tags were evaluated for accuracy and specificity using SAGEGenie from the National Cancer Anatomy Project^75^. Only tags with > 50% specificity were taken into account, and expression in retina, macula, and peripheral parts of the eye were evaluated, with comparison to brain and systemic tag expression. The Ocular Genomics Institute (Harvard, MA, USA) database for the retinal transcriptome^76^ integrated with UCSC Genome Browser, is comprised of RNAseq from three control retinas. It was used to determine if genes were present in retina, and to investigate presence of the coding/noncoding segments, and modifiers of those genes.

### Clinical Parameters and Genetics

Our investigation of clinical data was needed to ascertain if any clinical parameters affected gene expression. Multiple categories including gender, age, ethnicity, number of injections of anti-VEGF compounds provided, visual acuity, etc. were compared to gene expression via microarray or QPCR depending on the samples tested. Associations were evaluated using R with appropriate multivariate testing. Genotype for major AMD risk SNPs in the genes HTRA1, CFH, and C3 were also tested, and gene expression was compared between homozygote carriers for the wild type allele to those who carried a risk allele.

### Statistics

Statistics were performed with bioinformatics programs detailed above including R, InStat (GraphPad, La Jolla, CA, USA) and Excel (Microsoft, Redmond, WA, USA). Significance was determined via P-value < 0.05.

### Data Availability

The datasets supporting the conclusions of this article are available in GEO repository GSE76237 as well as in the additional supplementary data files referenced within this article.

## Additional Information

**How to cite this article**: Grunin, M. *et al*. Transcriptome Analysis on Monocytes from Patients with Neovascular Age-Related Macular Degeneration. *Sci. Rep*. **6**, 29046; doi: 10.1038/srep29046 (2016).

## Supplementary Material

Supplementary Information

Supplementary Table S1

Supplementary Table S2

Supplementary Table S3

Supplementary Table S4

Supplementary Table S5

Supplementary Table S6

Supplementary Table S7

Supplementary Table S8

Supplementary Table S9

Supplementary Table S10

Supplementary Table S11

## Figures and Tables

**Table 1:** Differential expressed genes (P < 0.05, log2 Fold Change (FC) > 1.5, RMA-normalized, ANOVA) between monocytes from nvAMD patients and controls.

Gene Number	79 Significant Genes in Microarray Analysis with log2 FC > 1.5		
	Transcript ID	P-value (nvAMD vs Control)	Log2 Fold-Change (nvAMD vs Control)
1	MS4A1	8.35E-04	−2.79
2	CD3G	2.38E-03	−2.28
3	LEF1	4.04E-03	−2.14
4	FAIM3	8.55E-04	−2.06
5	SNORD116-24	1.40E-03	−2.04
6	GZMK	2.12E-02	−1.92
7	KIR2DL3	2.55E-04	−1.91
8	SNORD116-8	7.31E-04	−1.91
9	SNORD116-15	4.23E-03	−1.89
10	IL7R	4.15E-02	−1.89
11	SLAMF6	1.37E-03	−1.87
12	KLRK1	7.44E-03	−1.87
13	SNORD116-5	2.70E-03	−1.86
14	CD24	1.94E-03	−1.86
15	SNORD116-14	3.93E-03	−1.85
16	KIR2DS2	2.53E-04	−1.84
17	PRF1	7.02E-03	−1.83
18	TGFBR3	7.26E-03	−1.81
19	CD2	9.23E-03	−1.80
20	KIR2DL1	4.45E-04	−1.77
21	FCER2	4.14E-03	−1.77
22	KIR2DS4	1.98E-04	−1.77
23	SNORD116-3	3.98E-03	−1.76
24	ZFY	3.76E-02	−1.76
25	KIR2DL2	3.52E-04	−1.76
26	IKZF3	5.66E-03	−1.76
27	CST7	6.46E-03	−1.76
28	KLRB1	2.00E-03	−1.75
29	CD3D	1.66E-02	−1.75
30	SKAP1	4.31E-03	−1.75
31	TCL1A	4.67E-03	−1.72
32	ITK	1.85E-02	−1.72
33	ETS1	1.19E-03	−1.72
34	P2RY10	2.48E-04	−1.71
35	CCR7	1.29E-02	−1.71
36	GPR56	7.58E-03	−1.70
37	KIR2DS1	2.98E-04	−1.70
38	GZMB	3.62E-04	−1.70
39	GZMA	1.43E-02	−1.70
40	CD79A	5.42E-04	−1.69
41	NKG7	2.87E-03	−1.69
42	CD8A	3.78E-03	−1.67
43	GNLY	4.39E-02	−1.67
44	SNORD94	4.32E-03	−1.66
45	CTSW	6.43E-03	−1.66
46	PAX5	1.82E-04	−1.66
47	SNORD116-20	1.10E-02	−1.66
48	FGFBP2	2.68E-02	−1.66
49	FCRLA	6.64E-04	−1.66
50	RNU5E	1.67E-02	−1.65
51	SNORD116-1	5.41E-03	−1.65
52	SNORD116-17	1.14E-02	−1.64
53	FCRL1	2.38E-03	−1.62
54	SH2D1A	1.17E-02	−1.61
55	CD5	2.07E-02	−1.61
56	SPOCK2	2.97E-02	−1.61
57	CD28	2.11E-02	−1.60
58	CD22	5.08E-03	−1.59
59	KIR3DL2	4.78E-04	−1.57
60	SNORA20	9.04E-03	−1.55
61	KIR3DS1	2.72E-03	−1.55
62	RASGRP1	2.85E-02	−1.55
63	CD247	2.20E-02	−1.54
64	SAMD3	2.44E-02	−1.53
65	CD96	3.58E-02	−1.53
66	NFATC2	9.54E-03	−1.53
67	RHOH	7.52E-03	−1.52
68	FCRL6	2.39E-02	−1.52
69	FCRL3	8.13E-03	−1.52
70	IFITM1	1.67E-02	−1.51
71	SNORA22	8.66E-03	−1.51
72	BTLA	1.22E-02	−1.51
73	IL2RB	3.64E-02	−1.51
74	SLED1	1.98E-02	1.60
75	FOSB	3.91E-02	1.61
76	OLR1	3.05E-02	1.66
77	MOP-1	2.76E-02	1.84
78	TMEM176B	1.33E-02	2.61
79	TMEM176A	2.23E-02	2.67

Log2 FC < 0 indicates downregulated in nvAMD, log2 FC > 0 indicates upregulated in nvAMD.
