# Supplementary material for: Transcriptome Analysis on Monocytes from Patients with Neovascular Age-Related Macular Degeneration
Source: Sci Rep. 2016 Jul 4;6:29046. doi: 10.1038/srep29046 (PMC4931446; doi:10.1038/srep29046)
Supplement: Supplementary Information [file srep29046-s1.doc]

**Supplementary Data Files**

Transcriptome Analysis on Monocytes from Patients with Age-Related Macular Degeneration

*Michelle Grunin^1^, Shira-Hagbi-Levi^1^, Batya Rinsky^1^, Yoav Smith^2^, Itay Chowers^1*^*

*^1^Department of Ophthalmology, Hadassah-Hebrew University Medical Center, Jerusalem, Israel. ^2^ Genomic Data Analysis Unit, Hebrew University, Jerusalem, Israel*

*For correspondence:

Itay Chowers, MD

Department of Ophthalmology

Hadassah-Hebrew University Medical Center

POB 12000, Jerusalem, Israel 91120

Tel: +972-50-857-3361

Fax: +972-2-6777228

Email: [chowers@hadassah.org.il](mailto:chowers@hadassah.org.il)

**Supplementary Material**: The following supplementary material is available with the online version of this paper.

**Supplementary Figures and Legends**

*Figure S1:*


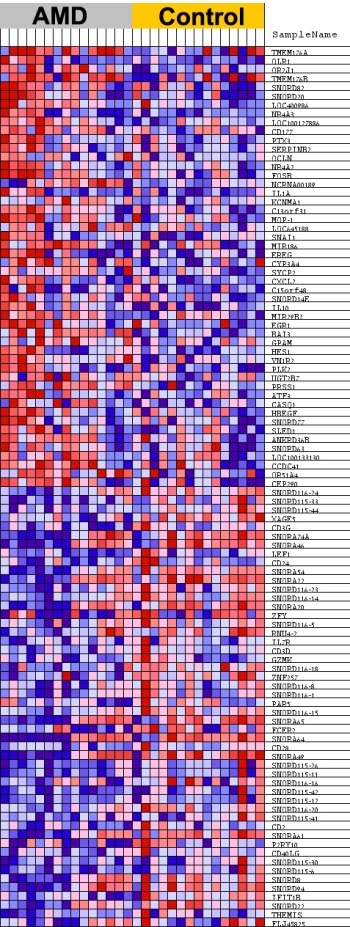


Figure S1: GSEA heat map indicating up (red) or downregulation (blue) between nvAMD patients and controls. This map is comprised of the top 50 up and top 50 down regulated genes according to the GSEA algorithm (P<0.05).

*Figure S2:
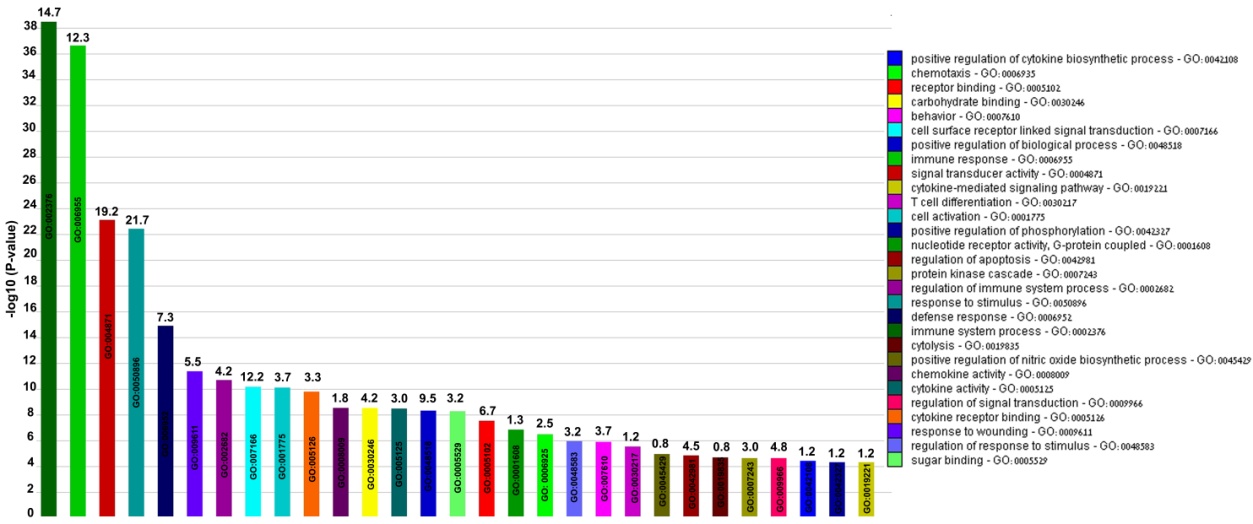
*

Figure S2: Functional analysis of differentially expressed genes in nvAMD monocytes. TANGO results for all 29 GO clusters from differentially expressed genes (N= 2,165, P<0.05). -log10 P-value is present as the Y axis, developed according to TANGO's bootstrap algorithm. The number on top of each cluster's column indicates percentage of genes from EXPANDER's microarray analysis present in the functional class cluster.

*Figure S3*:


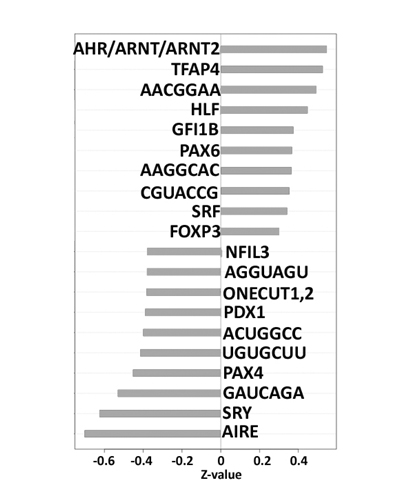


Figure S3: ISMARA z-score for transcription factors and microRNA whose motifs were enriched in genes upregulated and downregulated in AMD patients. Z-score>0 indicates upregulation in AMD, Z<0 indicates downregulation (P<0.05).

**Supplementary Tables and Legends**

*Table S1:* Genes which were differentially expressed between monocytes of nvAMD patients and controls (P<0.05, RMA-normalized, ANOVA). Data split in tabs of the spreadsheet by fold change (log2 FC): all (2,165 genes), log2 FC>1.2 (506 genes) and log2 FC>1.5 (79 genes). log2 FC<0 indicates downregulated in nvAMD, log2FC>0 indicates upregulated in nvAMD. A indicates nvAMD, while C indicates controls.

*Table S2:* Genes upregulated or downregulated between nvAMD patients and controls (P<0.05, RMA-normalized, EXPANDER algorithm). Cells highlighted in orange are genes that had an absolute log2 FC greater than 1.2 in RMA-normalized data, while cells highlighted in red are genes that had an absolute log2 FC greater or less than 1.5 in RMA-normalized data.

*Table S3:* Analysis with GSEAs algorithm for gene expression in monocytes according to microarray between nvAMD patients and controls. Positive score/FC indicates upregulation in nvAMD, negative score indicates downregulation in nvAMD. Cells highlighted in yellow are genes that were P<0.05 for the RMA-normalized data, Cells highlighted in orange are genes that had an absolute log2 FC greater than 1.2 in RMA-normalized data, while cells highlighted in red are genes that had an absolute log2 FC greater or less than 1.5 in RMA-normalized data for easy comparison between sets.

*Table S4:* Functional analysis according to the DAVID algorithm. The spreadsheet is split into multiple tabs: tab 1 indicates analysis on 2,165 genes (P<0.05), tab 2 indicates analysis on 506 genes (log2 FC>1.2, P<0.05), and tab 3 indicates analysis on 79 genes (log2 FC>1.5, P<0.05). Enrichment clusters (EC) are presented. Clusters highlighted in yellow indicate Benjamini-Hochberg FDR-P<0.05, Enrichment Score (ES)>2.

*Table S5:* Functional analysis according to the Enrichr algorithm. Results for 3 sets of genes: 2,165 genes with a P<0.05, 506 genes with P<0.05, log2 FC>1.2, and 79 genes with P<0.05, and log2 FC>1.5 are presented. Each tab indicates a separate algorithm result, with terms with adjusted P<0.05 highlighted in yellow. GO data, KEGG, Reactome and Proteome data is included for each gene set.

*Table S6*: GSEA MSigDB Gene List enrichment in genes upregulated in AMD (tab 1) or downregulated in AMD (tab 2). ES= Enrichment Score, NES= Normalized ES, P-value, Q-value and corrected values given along with leading edge rankings.

*Table S7*: GSEA uncurated look at enriched gene lists from the MSigDB, using all categories (C1-7: Positional Gene Sets, Curated Gene Sets, Motif Gene Sets, Computational Gene Sets, GO Gene Sets, Oncogenic Signatures, and Immunologic Signatures, accessible at http://software.broadinstitute.org/gsea/msigdb/collections.jsp). Tab 1 indicates the entire gene list (2,165 genes, P<0.05), tab 2 indicates log2 FC>1.2, tab 3 indicates log2 FC>1.5.

*Table S8*: Gene lists of genes upregulated and downregulated in MHC- and MHC+ mouse monocytes from the Immugen datasets. This data was compared to the microarray significant genes to compute overlap.

*Table S9*: Presence of SAGE tags corresponding to genes differentially expressed in nvAMD monocytes(P<0.05, log2 FC>1.5) at eye and other tissues. SAGE data was extracted from the National Eye Institute (NEI)(http://neibank.nei.nih.gov/EyeSAGE/index.shtml)

for genes TMEM176A, TMEM176B, FOSB, MS4A1, FAIM3, and OLR1 which were tested by QPCR. Macula (Mac), Peri (Periphery) and Retina all are listed, as are Body, Neural, and Eye.

*Table S10*: GSEA uncurated look at enriched gene lists from the MSigDB, using category 3, Motif Gene Sets (C3). Tab 1 indicates the entire gene list (2,165 genes, P<0.05), tab 2 indicates log2 FC>1.2, tab 3 indicates log2 FC>1.5.

*Table S11*: HOCOMOCO database and MEME results for transcription factor (TF) motifs enriched in differentially expressed genes (log2 FC>1.5, P<0.05) (tab 1, 79 genes) or log2 FC>1.2 (tab 2, 506 genes). *Italics* indicate TF which was found in both nvAMD and controls, eliminating it from results. **Bold**+ *Italics* indicates that TF was found to be enriched in data upregulated in nvAMD both in comparison with nvAMD vs Controls and in genes upregulated in nvAMD vs a randomized shuffle. **Bold**+ Underline indicates that TF was found to be enriched in data downregulated in nvAMD both in comparison with nvAMD vs Controls and in genes downregulated in nvAMD vs a randomized shuffle. Corrected P-value performed according to FDR.
